# Influence of internet exposure on sexual behaviour of young persons in an urban district of Southwest Nigeria

**DOI:** 10.11604/pamj.2016.25.261.2630

**Published:** 2016-12-30

**Authors:** Oyedunni Sola Arulogun, Ifeyinwa Arinze Ogbu, Isaac Oluwafemi Dipeolu

**Affiliations:** 1 Department of Health Promotion and Education, Faculty of Public Health, College of Medicine, University of Ibadan, Ibadan, Nigeria; 2 Family Health International (FHI 360), Monitoring & Evaluation Department, Plot 1073 A-1, Godab Plaza J.S. Tarka Street, Area 3, Garki Abuja, Nigeria

**Keywords:** Internet use, young persons, pornographic sites, sexual behaviour

## Abstract

**Introduction:**

The proportion of young people exposed to pornographic materials through the internet in Nigeria is increasing. However, the influence of the exposure on their sexual behaviour has not been fully explored. This study therefore explored the effects of internet exposure on the sexual behaviour of young persons in Ibadan North Local Government Area of southwest Nigeria.

**Methods:**

A survey of 413 young persons was done using a pretested self-administered questionnaire which included questions on internet exposure and its influence on behaviour. Data were analysed using descriptive statistics, Chi-square test and logistic regression.

**Results:**

Mean age of males was 21.7 ± 3.4 years while that of females was 20.9 ± 3.2 years. Forty-nine percent of the respondents used the internet for the first time between the ages of 15-19 years. Main source of information about the internet was friends (63.3%) and 99.3% accessed the internet from cybercafé. Seventy-two percent had ever stumbled on pornographic sites. Reactions included glancing through before closing (45.2%), closure of the sites (38.5%), and minimizing page to view later (12.5%). Post-exposure influence on behaviour included engagement in oral sex (48.3%), body tattoo (18.3%), having multiple sexual partners (11.6%) and homosexuality (5.0%). More males (95% CI OR =1.245-6.465) and frequent users (95% CI OR =1.168-3.497) were likely to report a change in sexual behaviour.

**Conclusion:**

Internet use was common among the young persons. Interventions aimed at reducing exposure to sexual content on internet targeting young persons especially the males and cybercafé operators are advocated.

## Introduction

The Internet is the core of computer mediated communication. It is worldwide and connects millions of computer networks, providing an incredible array of information adolescents can access [1] and because of its fluid capacities, the Internet has more up-to-date information than books. Young people throughout the world are increasingly using the Internet, despite substantial variation in use in different countries around the world and in socioeconomic groups. The key concern about the health of young people is the extent to which they have access to resources that promote their development. This is strongly linked to early concerns about the way children and teenagers use the Internet which is suspected to have negative side-effects [2, 3]. Other concerns as documented by Fleming et al [4] included teenagers’ exposure to inappropriate content. One major emerging worrisome dimension in cyberspace is pornography in its various guises the proliferation and mainstreaming of which had reportedly influenced youth culture and adolescent development in unprecedented and diverse ways [5–7]. In addition, Flood [8], Häggström-Nordin, Sandberg, Hanson and Tydén [9] and Wolak, Mitchell and Finkelhor [10] have documented that Internet-enabled devices have indiscriminately allowed people of all ages to encounter, consume, create, and distribute sexually explicit content with growing evidence that these phenomena are increasingly common among adolescents worldwide. This has made intentional or accidental viewing of pornographic materials online to be on the increase. Although empirical data about Internet pornography and its impact on the life of the teeming youth and children in Nigeria are not readily available, the fact that 32% of Internet users in Nigeria are children and teenagers of age range 7 to 18 years is an important fact that is worthy of note. Fears are rife in some sectors about the possible negative consequences obnoxious and uncensored Internet contents will have on the psychosocial well-being of varying categories of users and especially children and teens in Nigeria [11].

Several studies have been done on the use of Internet in Africa including that of Ojedokun [12] who studied use of the Internet by students of the University of Botswana. His study revealed that 77% of the respondents had used Internet. Ajuwon [13] studied Internet use by first year clinical and nursing students of the University College Hospital in Ibadan, Nigeria and found that 60% of the respondents had used Internet. Odusanya and Bamgbala [14] found that 58% of the medical and dental students in their final year at the University of Lagos, Nigeria whom they studied had used the Internet. Given that so many adolescents are spending so much time on the Internet, it is important that awareness of the impact of its contents on adolescent behaviour, well-being, and development be made known. According to the prediction of social learning theory, teens exposed to certain unconventional behaviours can adopt and internalize such behaviours as conventional ones. Given the popularity of the Internet amongst young persons, some researchers have investigated the relationship between young persons' involvement with online sexual activities (including online chats, meeting partners, and looking for romantic and sexual relationships) and the development of their sexuality. Cooper et al [15] found that excessive usage (as measured by time spent in viewing sexually related activities online) was positively related to stress and sexual sensation seeking. Similar study conducted by Goodson et al [16] in Adebayo et al [17] among university students, found that respondents' attitude towards seeking sex information and sexual entertainment varied based on the frequency of their Internet usage. Despite the rising level of Internet usage particularly among young people in Nigeria, few studies have examined the effects that sites with explicit sexual contents may have especially on the sexual inclination and behaviour of young persons. This study therefore was carried out to determine the influence of Internet use on sexual behaviour of adolescent in Ibadan North Local Government Area, an urban district of Ibadan metropolis, southwest Nigeria.

## Methods

**Research design:** This descriptive and cross-sectional study aimed at documenting the influence of exposure to Internet on the sexual behaviour of young persons. It sought to identify the prevalence of Internet use, activities engaged in and its effect on sexual behaviour.

**Study setting:** Ibadan North Local Government Area (LGA) constitutes the study setting. It is one of the five LGAs in Ibadan metropolis and was created on 27th September 1991 out of the defunct Ibadan Municipal Government. The LGA comprises of 12 political wards with a multi-ethnic population. There are numerous educational institutions in the LGA such as the University of Ibadan, The Polytechnic Ibadan, 78 public and 48 private primary schools as well as 80 public and 20 private secondary schools. There are quite a number of cybercafés all over the LGA and are of various sizes from city centre to small streets. Majority of these cybercafés are concentrated around the educational institutions in Agbowo, Polytechnic and Bodija areas of the LGA.

**Sampling procedure and sample size:** A four-stage sampling technique which comprised of stratified, proportionate and simple random sampling techniques was adopted in the selection of the 413 young persons from households in the local government areas. In the first stage, five wards were selected out of twelve wards in Ibadan North LGA, secondly, five streets were randomly selected in each of the five wards selected for the study in Ibadan North LGA. In stage three, household was systematically selected within the selected streets for study while stage four involved the selection of 413 eligible respondents from households.

**Instrument for data collection:** A pretested self-administered semi-structured questionnaire which contained questions on overall activity and practices related to Internet use, pornography exposure and behaviour change after exposure was used for data collection.

**Data collection process:** Each interview started with an introduction and overview of the research including the objectives of the study. The respondents were told not to write any name on the self-administered questionnaire. Respondents were encouraged to ask questions on what they do not understand in the questionnaire. Explanations were given to respondents as required to aid their understanding of unfamiliar terms. The questionnaires were retrieved back from each respondent immediately after completion and they were reviewed for completeness.

**Data management, analysis and presentation:** Administered copies of the questionnaire were edited and coded with the aid of a coding guide. Coded data were entered into a computer for analysis using the IBM/Statistical Package for Social Sciences (SPSS) version 15.0. Summary statistics such as means, median and standard deviations were used to summarize quantitative variables. Association between categorical variables was tested using the Chi square test. Logistic regression analysis was done to identify significant predictors of two dependent variables: action taken on experiencing pornographic material and reported change in sexual behaviour. Level of significance was at p=0.05.

**Ethical consideration:** The study followed the ethical principles guiding the use of human participants in research. Prior to entering the study site, permission to carry out the study was obtained from relevant community gate keepers and from parents of the youth concerned. All respondents were informed that participation in the survey was voluntary, and that they may not to participate if they choose to or could withdraw from participating at any time. Respondents were assured that confidentiality of responses would be maintained during and after data collection. Only numbers were assigned to facilitate data entry and analysis and no one can link the identity of the respondents with the assigned numbers. Verbal informed consent was obtained from each respondents before a copy of the questionnaire was given to them.

## Results

### Socio-demographic characteristics of the respondents

The distribution of respondents' socio-demographic characteristics revealed that about two-thirds were between 20 to 24 years, 29.8% were less than 20 years and 6% did not indicate their age. There were more males (70.5%) than female respondents (28.6%). The highest proportion of respondents had tertiary education (60.3%) followed by senior secondary (23.5%), junior secondary (1.2%) and primary (0.2%) education. The proportion of out-of-school respondents was 13.1%. They were predominantly Yoruba (76.5%) followed by Igbo (18.4%), Hausa (2.4%) and others (1.5%). There were more Christians (83.8%) than Muslims (14.8%).

### Internet use

About half (49.2%) of respondents started using the Internet between 15-19 years and 99.3% access the services from the cybercafés. Main source of information about the Internet was friends (63.3%). Frequency of use showed that 29.5% access the Internet every day. Duration of time spent online ranged from 30 minutes to three hours (Table 1). Main activities engaged in included sending or reading mails (55%), on line chatting (34.1%), research/homework (31%), information about current events (27.6%), information about schooling abroad (24.9%), music downloads (18.6%), job search (16.2%) and playing online games (12.6%). Visiting pornographic sites was reported by 8.0% and 3.6% sought information on health issues.

**Table 1:** Frequency and duration of Internet use by respondents

Frequency of Daily Internet use	Percentage
Once	62.7
2 – 3 times	17.2
More than three times	12.1
No response	8.0
**Frequency of Weekly Internet use**	
Daily	29.5
Once a week	29.5
2-3 times a week	24.9
5 times a week	9.5
No response	6.1
**Time spent during each visit**	
30 minutes – 1 hour	53.0
2-3 hours	29.1
More than 3 hours	14.5
No response	3.4

### Respondents visit to pornographic sites and their reaction

Table 2 shows the proportion of adolescents who had visited or stumbled on pornographic sites and their reactions. Sex differentiation was noticed in the reactions to the sites: glancing through before closing 45.2% (females, 30.1%; males, 46.7%), closure of the sites 38.5% (females, 57.5%; males, 38.7%), and minimizing page to view later 12.6% (females, 12.3%; males, 13.6%). On whether respondents discussed what they viewed on internet, 55.2% of respondents never discussed pornographic scenes viewed with anybody, 32.6% discussed with friends of same sex, 9.6% shared the experiences with friends of the opposite sex and only 2.6% discussed with parent/guardian.

**Table 2:** Proportion of respondents who had ever visited or stumbled on pornographic sites and their reactions

Ever visited or stumbled on pornographic site	Percentage
Yes	65.4
No	34.6
**Frequency of visit or stumble (N=270)**	
Regularly	20.4
Occasionally	79.6
**Reactions on stumbling or visiting site**	
Glance through and close	45.2
Close immediately	38.5
Minimize and view later	12.6
No response	3.7
Person respondent shares experience on site with	
Nobody	55.2
Friend (same sex)	32.6
Friend (opposite sex)	9.6
Parents/guardian	2.6

### Influence of exposure to sexually explicit sites on sexual behaviour

**Figure 1:**
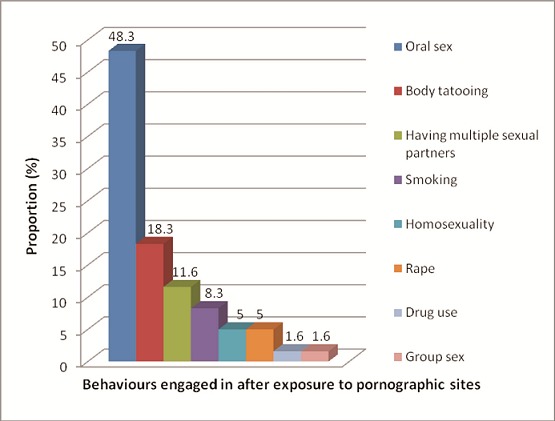
Behaviours engaged in after exposure to pornographic sites

Changes in sexual behaviour were reportedly observed by 31.1% of respondents following exposure to sexually explicit sites and 19.5% practiced what was seen. Practices engaged in post-exposure included oral sex (48.3%), body tattoo (18.3%), having multiple sexual partners (11.6%) and homosexuality (5.0%) (Figure 1). Daily users (95% CI OR = 1.168 - 3.497) and males (95% CI OR =-1.245 - 6.465) were more likely to visit pornographic sites compared with other respondents.

## Discussion

The high prevalence of Internet use among males suggests that males are more inclined to technology than females a trend that needs to be reversed. High number of respondents accessing Internet from cybercafé is not unexpected considering the unstable electricity supply in the country as the cybercafés have alternative source of power supply. In addition, going to cybercafé also provides avenue for social networking which agreed with the finding that friends were the main source of information about Internet. In this study, more than half of respondents who ever had online chatting admitted discussing relationships with strangers. This trend is increasing and has assumed a sad dimension in recent times in Nigeria. This has to be reversed if these young persons are to be protected and shielded from unscrupulous persons. Accessing health information was the least mentioned activity engaged in on-line. This may not be unconnected with the not youth-friendly nature of the sites. Most of the sites present information using medical terminologies which may not be simple enough for easy understanding. This may account for the large number who engages in chatting, reading and sending mails as documented by Osakinle [18].

Most of the young persons interviewed did not share experiences they had on the Internet with anybody and the few that did so mostly with their friends. This need to be addressed so that the amount of misinformation and/or misconceptions among young persons as it relates to sexuality issues are reduced to the barest minimum. Poor parent-child communication was also reported in this study. This may not be unconnected to the fact that parents are not well equipped to discuss such issues with their young ones which is fuelled by cultural norms surrounding sexual and reproductive health issues. For parents to be able to reach out to their young persons, their capacity needs to be strengthened in that area.

Furthermore, this study affirms previous documentation that males report more positive attitude toward pornography from an early age than females [19, 20]. Frequency of Internet use was significantly associated with practice of content of sexually explicit sites. This corroborates earlier findings that sexual behaviour can be acquired through exposure to pornography and sexual models on the Internet through imitating and copying of such acts [17, 21, 22]. A significant association was recorded between action taken on exposure to pornographic materials on the Internet for gender and frequency of Internet use. Females compared to males were more likely to react negatively to such exposure and more frequent users were more likely visit pornography sites intentionally. This gender difference in action taken on exposure to pornography corroborates earlier studies [17, 23, 24]. Also, frequency of Internet use was significantly associated with practice of the content of sexually explicit sites. This degree of association has been reported by Inyang [20], Egbochukwu et al [25] Odeyemi et al [22] and Brown et al [26].

The results of the logistic regression analysis revealed that daily users were more likely to view pornography site than other respondents and males more likely than females to experience change in sexual behaviour. This is consistent with previous studies that have found gender and Internet use to be predictive of sexual attitude and behaviour orientation of young adults [17, 27].

## Conclusion

This study documents a high prevalence of Internet use among young person. It also documented an association between frequent Internet use and permissive sexual behaviour highlighting Internet use as a significant predictor of adolescent sexual behaviour. In addition, poor parent-child communication was re-affirmed. Therefore, if the full educational potential of the Internet is to be realized for young persons, a multi-pronged intervention targeting young persons, building the capacity of parents for improved communication and instituting stringent guidelines for the operation of cybercafé is necessary.

### What is known about this topic

- Previous studies have shown that there is high prevalence of Internet use among young person;
- Previous documentation show that males report more positive attitude toward pornography from an early age than females. These studies found gender and Internet use to be predictive of sexual attitude and behaviour orientation of young adults;
- In addition, findings from previous studies show that frequency of Internet use was significantly associated with practice of content of sexually explicit sites. Thus sexual behaviour can be acquired through exposure to pornography and sexual models on the Internet through imitating and copying of such acts.

### What this study adds

- Level of education was not a barrier to accessing the Internet;
- Most of the young persons interviewed did not share experiences they had on the Internet with anybody and the few that did, did so mostly with their friends;
- About a third reportedly experienced behaviour change after exposure to sexually explicit sex and almost a fifth of respondents actually practiced what was seen on the sites.
